# Perceptions of the impact of disability and impairment on health, quality of life and capability

**DOI:** 10.1186/s13104-019-4324-y

**Published:** 2019-05-24

**Authors:** Nathan Bray, Rhiannon Tudor Edwards, Luke Squires, Valerie Morrison

**Affiliations:** 1 School of Health Sciences, Bangor University, Fron Heulog, Bangor, Gwynedd LL57 2EF UK; 2 Centre for Health Economics and Medicines Evaluation, Bangor University, Ardudwy Hall, Bangor, Gwynedd LL57 2PZ UK; 3 School of Psychology, University of Liverpool, Whelan Building, Brownlow Hill, Liverpool, L69 3GB UK; 4 School of Psychology, Bangor University, Adeilad Brigantia, Penrallt Road, Bangor, Gwynedd LL57 2AS UK

**Keywords:** Disability, Health psychology, Quality of life, Wellbeing, Health-related quality of life, Capabilities

## Abstract

**Objective:**

The impact of impairment and disability on quality of life can be considerable, however advances in assistive technology can help to optimise physical and psychosocial functioning. Little is known about how impairment and subsequent adaptation influences health state perceptions, particularly amongst the general public. The aim of this pilot project was to examine student perceptions of what it would be like to live with a physical or sensory impairment, and how adaptation influences health and quality of life.

**Results:**

In total 151 undergraduate Psychology students were invited to participate in a questionnaire-based survey. Ethical approval was granted by an academic ethics committee. The survey included a range of validated outcome measures relating to illness perceptions and quality of life, including the B-IPQ, EQ-5D-3L and ICECAP-O. Surveys were divided into two parts: firstly, participants were asked to self-report their own health; and secondly participants were asked to estimate the health impacts of a range of hypothetical states of impairment. Severe adapted impairments were perceived to have less impact on health status than moderate un-adapted impairments. Hearing impairment was perceived to have the least impact on health status, whilst mobility impairment was perceived to have the largest impact on health status.

**Electronic supplementary material:**

The online version of this article (10.1186/s13104-019-4324-y) contains supplementary material, which is available to authorized users.

## Introduction

Technological advances in health and social care have led to a plethora of assistive technologies to enable people with impairments or disabilities to ameliorate their impact, to varying extents. Despite the growing availability of such devices, there is still relatively little known about how individual’s perceive the use of assistive technology—devices designed to improve self-management, but which may increase impairment or disability visibility for example [1, 2].

The perception of assistive technologies and their impact on health and quality of life is particularly important in the context of National Health Service (NHS) funding, where population preferences for different health states play a role in funding decisions. These preferences are measured using preference-based measures (PBMs) of health-related quality of life (HRQoL), such as the EQ-5D, and used to calculate quality-adjusted life years (QALYs). In recent years the National Institute for Health and Care Excellence (NICE) has promoted the principle of cost-effectiveness, operationalising opportunity cost in terms of cost per QALY estimates to guide NHS expenditure and commissioning [3].

The aim of this pilot project was to examine young adult (student) perceptions of what it would be like to live with an impairment or disability, and the potential influence of adaptation on perceived health and quality of life. The objective was to establish the methods and measures to be used in a larger study of lay perceptions of impairment, disability, quality of life and disability ‘costs’ in a wider general public sample, and ultimately a study of these perceptions amongst individuals with impairments and/or disabilities.

## Main text

### Methods

#### Recruitment and data collection

A questionnaire-based survey design was used to capture data. First and third year undergraduate students were invited to participate in the study as part of an undergraduate-level Psychology course at Bangor University. Participants received credits relating to their course for completing the survey.

#### Measures

The questionnaire addressed a range of demographic questions, illness perceptions (Brief Illness Perceptions Questionnaire [B-IPQ]) [4] and quality of life outcome measures, including the EuroQoL 5-Dimension 3-Level (EQ-5D-3L) questionnaire and Visual Analogue Scale (EQ-VAS) [5] and the ICEpop CAPability measure for Older people (ICECAP-O) [6]. The EQ-5D is a generic, validated HRQoL measure recommended by NICE for QALY calculation [3]. The EQ-5D was analysed to produce an index score between 0 (state of death) and 1 (perfect health).

The EQ-VAS is a self-rated measure of health status; respondents are asked to indicate their health on the day of completion using a scale ranging from 0 (worst imaginable health) to 100 (best imaginable health).

The ICECAP-O is a validated capability measure which focuses on wellbeing beyond health. It is scored from 0 (no capability) to 1 (full capability). The ICECAP-O is designed for use specifically with older adults. The generic adult version of the ICECAP measure was unavailable at the time of undertaking this research, therefore the ICECAP-O was used as an approximate measure of capability.

The B-IPQ is a nine-item scale measuring cognitive illness representations (i.e. consequences, timeline, personal control, treatment control, and identity), emotional representations (i.e. concern and emotions) and illness coherence. Each item is scored on a Likert scale from 0 to 10 (with 10 representing a more threatening perception). A computed overall score indicates the degree to which an illness is perceived to be threatening; the higher the score, the more threatening. The B-IPQ is typically scored as distinct subscales, thus the subscales were summed in the direction of illness severity/negative perceptions.

#### Survey design

Data were collected primarily through an online survey. Surveys were divided into two separate parts: firstly, participants were asked to self-report their own health; and secondly participants were asked to estimate the health impacts of a range of hypothetical states of impairment.

Participants self-reported HRQoL (using the EQ-5D), health status (using the EQ-VAS) and capability (using the ICECAP-O); referred to as ‘personal’ scores in the results. For the hypothetical states of impairment participants estimated health status (using the EQ-VAS), capability (using the ICECAP-O) and illness perception (using an adapted B-IPQ); referred to as ‘perceived’ scores in the results.

The content of hypothetical states of impairment varied depending on the measure:

- ICECAP-O: participants asked to estimate capability associated with a generic state of impairment (i.e. sensory or physical impairment).
- B-IPQ: participants asked to estimate illness perceptions for two specific states of impairment: mobility impairment related to Multiple Sclerosis and visual impairment related to Age-Related Macular Degeneration. Given that the causes of the hypothetical states of impairments were unknown to participants, the causal item of the B-IPQ was removed to remain valid and to not encourage misattribution in participants.
- EQ-VAS: participants asked to estimate health status for 12 states of impairment, grouped into three broad categories: visual impairment, hearing impairment and mobility impairment. Each category of impairment was divided into four states: moderate; moderate with adaptation (e.g. use of a mobility aid); severe; and severe with adaptation.

The differentiation of the content of the measures was chosen to reduce burden on respondents. Participants were asked to complete the measures for the hypothetical states of impairment according to the perceived impact on health, wellbeing and quality of life. Wording of the hypothetical states is presented in Additional file 1: Appendix 1.

#### Analysis

Descriptive statistics were produced using Excel. Personal mean scores for the EQ-5D, EQ-VAS and ICECAP-O were compared to population norms to give an indication of the comparability of the sample to the wider population. The EQ-VAS was used to examine the role of adaptation and perceived severity of impairment on health status, with participants asked to indicate the relative health state value of different hypothetical states of impairment. The ICECAP-O mean total scores and individual item mean scores were compared between perceived and personal outcomes to examine differences in how the sample perceived their own capability and that of a generic state of impairment. The B-IPQ subscales examined which aspects of illness perception had the greatest impact on lay perceptions of the threat of mobility and visual impairments on health.

#### Sample

In total 151 participants were recruited. The average age of participants was 20 years (SD 3.03). Completeness of data depended on each measure, varying from 149 participants (EQ-5D) to 151 (EQ-VAS, ICECAP-O, B-IPQ). Overall, the level of missing data was therefore relatively small. Inclusion and exclusion criteria were not explicitly stated as convenience sampling was used, although all participants had to be aged 18 or over and able to provide informed consent.

### Results

#### Self-reported HRQoL, health status and capability: comparison to norms

See Table 1 for all self-reported outcomes. The mean self-reported HRQoL score for participants was 0.91 (SD 0.15), which although lower than the UK population norm score for under 25 year olds (0.94; [7]), still represents a high mean HRQoL score. The mean EQ-VAS score for the sample was 79.65 (SD 16.29), equating to an average self-reported health status approximately 9% below the UK population norm for this age group (88.68; [7]).

**Table 1:** Outcome measure total scores: personal and perceived for different states of impairment

	Mean	SD	Median	Range	N
Personal					
EQ-5D	0.912	0.147	1	0.159 to 1	149
EQ-VAS	79.65	16.29	82	20 to 100	151
ICECAP	0.848	0.108	0.870	0.362 to 1	151
Perceived					
ICECAP GI	0.678	0.173	0.699	0 to 1	150
B-IPQ MI	52.81	8.04	53	28 to 71	150
B-IPQ SI	55.86	9.22	56	27 to 78	151

*GI* generic impairment, *MI* mobility impairment, *SI* sensory impairment

An exact population norm for the ICECAP-O was not available, however an approximated mean has been reported as 0.83 [8], slightly below the sample self-reported mean of 0.85 (SD 0.11).

#### Perceived health status of hypothetical states of impairment

**Fig. 1:**
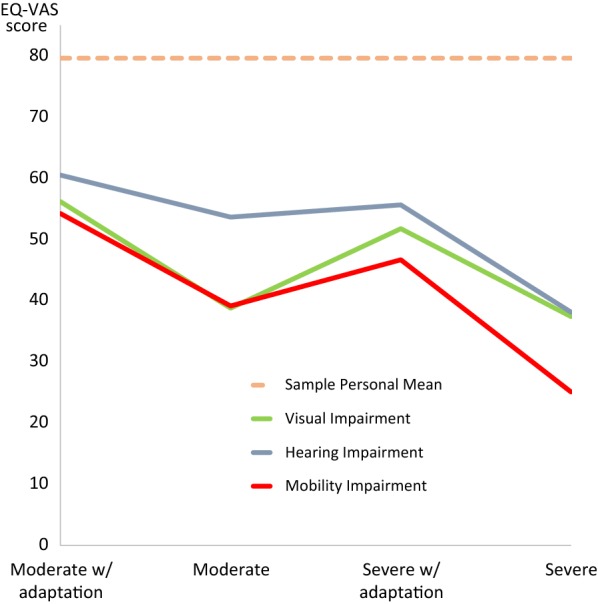
Perception of EQ-VAS health status in states of visual, hearing and mobility impairment, and the effect of adaptation

Moderate adapted impairments were perceived to have the least impact on health status, with EQ-VAS mean scores ranging from 54.24 (SD 20.93) for visual impairment to 60.51 (SD 19.50) for hearing impairment (see Additional file 2: Table S1). Severe adapted impairments (ranging from 46.68 to 55.65) were perceived to have less impact on health status than moderate un-adapted impairments (ranging from 38.77 to 53.65) across all three categories of impairment (see Fig. 1). Hearing impairment was perceived to have the least impact on health status (ranging from 38.12 to 60.51), whilst mobility impairment was perceived to have the largest impact on health status (ranging from 25.08 to 54.24).

#### Perceived capability of impaired health states

**Fig. 2:**
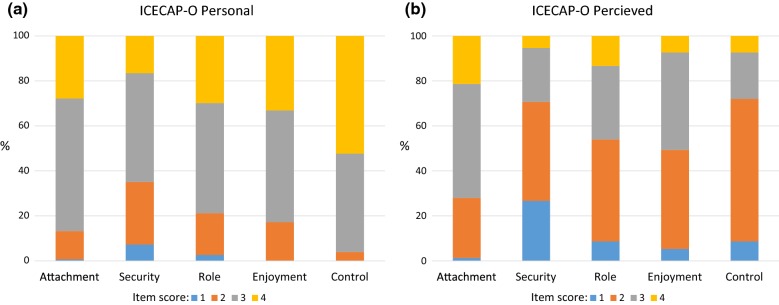
Distribution of individual item scores on the ICECAP-O. **a** Distribution of individual item scores on the ICECAP-O for personal wellbeing. **b** Distribution of individual item scores on the ICECAP-O for perceived wellbeing

The mean perceived ICECAP-O score for a state of generic impairment (physical or sensory) was 0.68 (SD 0.17), 17% less than the sample personal mean, indicating that the sample generally perceived states of impairment to have a detrimental impact on capability. The largest difference in mean individual item raw score on the ICECAP-O between personal and perceived results was for the Control item (3.48 and 2.27 respectively). See Fig. 2 and Additional file 3: Table S2.

#### Illness perception for mobility impairment and visual impairment

The sample perceived mobility and visual impairments to be highly threatening to health and wellbeing, with mean B-IPQ scores of 52.81 and 55.86 respectively on a scale from 0 to 80 (80 representing the most threatening view of illness; e.g. low control, high concern). For both B-IPQ hypothetical states of impairment, participants scored the Timeline and Concern items the highest (i.e. chronic timeline, high concerns) and the Treatment Control and Coherence items the lowest on average (see Additional file 4: Table S3 and Additional file 5: Table S4).

### Discussion

The results from this study indicate that young able-bodied individuals perceive a difference in the health impacts of adapted and un-adapted states of impairment. Methods of applying quality of life weights to health benefits, as recommended by NICE through the calculation of QALYs and capabilities, may not fully account for the impact of adaptation and assistive technology on health and quality of life, and potentially how the public perceptions of HRQoL may differ with and without such adaptations.

NICE stipulate that in the development of PBMs for QALY calculation, representative general population samples should be used to value health states [9]. The justification is that societal resources should be allocated in a way that is relevant to the general population, particularly in a publicly funded health care system [9]. However, when assessing the desirability of hypothetical health states, individuals focus on the transition from their own health state to the hypothetical health state, thus general public beliefs about the impact of disabilities do not always reflect the lived experience [10, 11]. Focus on personal transition means that processes such as adaptation are not accounted for, causing uncertainty in how states of disability impact outcomes [12].

Due to the underlying trade-off between quantity and quality of life in the calculation of QALYs, there is a tendency for lower value to be placed on extending the length of life of people with long-term disabilities and impairments [13], as their quality of life is routinely considered to be worse than that of an able-bodied person. Thus, when using the QALY framework to assess the outcomes of individuals with long-term impairments, it is difficult to achieve substantially higher quality of life when compared to individuals without impairments, raising concerns about bias [14].

One of the underlying issues is that the definition of HRQoL differs profoundly between people with disabilities and the general public [15]. For instance, when asked to define HRQoL, young wheelchair users focus on a number of concepts not explicitly measured using generic PBMs, such as ability to adapt, achievement and independence [16]. The experience of disability also affects HRQoL perceptions—mechanisms of adaptation, coping and adjustment can help individuals with disabilities to experience diminishing effects to their HRQoL over time [17]. The evaluation of states of disability by non-disabled individuals may therefore cause such states to have an exaggerated perceived impact on HRQoL and health status [18], as these processes of adaptation and adjustment are not accounted for using generic PBMs. Condition specific approaches to QALY calculation may therefore be more sensitive, although less comparable.

### Conclusion

To summarise, participants rated hypothetical states of impairment to be between 19 and 55% lower than their own health states in terms of EQ-VAS health status. Likewise, participants rated levels of capability in a broadly defined state of impairment to be 17% lower than their own self-rated capability. Participants perceived mobility and visual impairments to be highly threatening to health and wellbeing although adapted states were perceived to have less impact on health status than un-adapted states. Furthermore participants scored severe adapted health states higher than moderate un-adapted heath states. Hearing impairment was perceived to have the least impact on health status, whilst mobility impairment had the highest impact.

## Limitations

As recruitment was based on a convenience sample of undergraduate students, it is unlikely that the results are representative of the wider population, thus further research is needed to determine the perceptions of society more generally. In order to reduce burden on participants, the range of hypothetical health states presented to participants differed according to each outcome measure; however comparability across measures would have been easier with a more uniform approach to survey design.

## Additional files

**Additional file 1: Appendix 1.** Study questionnaire. Example of the study questionnaire used for data collection; the questionnaire includes all hypothetical states used for perceived health state analyses.

**Additional file 2: Table S1.** Perception of EQ-VAS health status in states of visual, hearing and mobility impairment, and the effect of adaptation. This table shows the effect of adaptation on the perceived health status (measured using EQ-VAS) of different states of visual, hearing and mobility impairment.

**Additional file 3: Table S2.** Individual item score proportions (%) on the ICECAP-O (personal and perceived results). This table shows the difference between perceived (i.e. a hypothetical state of generic impairment) and personal item scores on the ICECAP-O.

**Additional file 4: Table S3.** Individual item score proportions (%) on the B-IPQ (mobility impairment scenario). This table shows perceived individual item scores on the B-IPQ for a hypothetical state of mobility impairment.

**Additional file 5: Table S4.** Individual item score proportions (%) on the B-IPQ (visual impairment scenario). This table shows perceived individual item scores on the B-IPQ for a hypothetical state of visual impairment.

## Data Availability

The datasets generated and/or analysed during the current study are available from the corresponding author on reasonable request.

## Abbreviations

B-IPQ: Brief Illness Perceptions Questionnaire
EQ-5D: EuroQoL 5-Dimension
EQ-5D-3L: EuroQoL 5-Dimension 3-Level
EQ-VAS: EuroQoL Visual Analogue Scale
GI: generic impairment
HRQoL: health-related quality of life
ICECAP-O: ICEpop CAPability measure for Older people
MI: mobility impairment
NHS: National Health Service
NICE: National Institute for Health and Care Excellence
PBM: preference-based measure
QALY: quality-adjusted life year
SD: standard deviation
SI: sensory impairment
