# Supplementary material for: Perceptions of the impact of disability and impairment on health, quality of life and capability
Source: BMC Res Notes. 2019 May 24;12:287. doi: 10.1186/s13104-019-4324-y (PMC6534923; doi:10.1186/s13104-019-4324-y)
Supplement: Supplementary file 1 — Additional file 1: Appendix 1. Study questionnaire. Example of the study questionnaire used for data collection; the questionnaire includes all hypothetical states used for perceived health state analyses. [file 13104_2019_4324_MOESM1_ESM.doc]

**
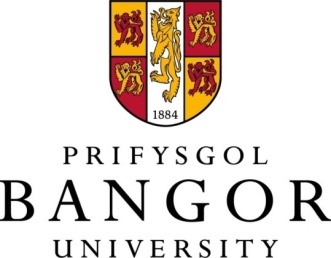
**

**PERCEPTIONS OF DISABILITY**

School of Psychology

Centre for Health Economics and Medicines Evaluation

Bangor University

DRAFT February 2013

Contents

A Your own health

B Perceptions of disability

C Perceptions of disability thermometer

D Perceptions of disability with adaptations for daily living

E The NHS and disability

F General questions

**A Your own health**

By placing a tick in one box in each group below, please indicate which statements best describe your own health state today

**Mobility**

I have no problems in walking about ❑

I have some problems in walking about ❑

I am confined to bed ❑

**Self-Care**

I have no problems with self-care ❑

I have some problems washing or dressing myself ❑

I am unable to wash or dress myself ❑

**Usual Activities** (e.g. work, study, housework, family or leisure activities)

I have no problems with performing my usual activities ❑

I have some problems with performing my usual activities ❑

I am unable to perform my usual activities ❑

**Pain/Discomfort**

I have no pain or discomfort ❑

I have moderate pain or discomfort ❑

I have extreme pain or discomfort ❑

**Anxiety/Depression**

I am not anxious or depressed ❑

I am moderately anxious or depressed ❑

I am extremely anxious or depressed ❑

**To help people say how good or bad a health state is, we**

90

80

70

60

50

40

30

20

10

100

**Worst imaginable health state**

0

**Best imaginable health state**

**have drawn a scale (rather like a thermometer) on which**

**the best state you can imagine is marked 100 and the**

**worst state you can imagine is marked as 0.**

**We would like you to indicate on this scale how good or bad your own health is today, in your opinion. Please do this by drawing a line from the box below to whichever point on the scale indicates how good or bad your health state is today.**

**Your own health state today**

**ABOUT YOUR QUALITY OF LIFE**

By placing a tick in ONE box in EACH group below, please indicate which statement best

describes your quality of life at the moment.

1. Love and Friendship

4

3

2

1

I can have all of the love and friendship I want

I can have a lot of the love and friendship I want

I can have a little of the love and friendship I want

I cannot have any of the love and friendship I want

1. Thinking about the future

4

3

2

1

I can think about the future without any concern

I can think about the future with only a little concern

I can only think about the future with some concern

I can only think about the future with a lot of concern

**Tick**

**one**

**box**

**only in**

**each**

**section**

1. Doing things that make you feel valued

4

3

2

1

I am able to do all of the things that make me feel valued

I am able to many of the things that make me feel valued

I am able to do a few of the things that make me feel valued

I am unable to do any of the things that make me feel valued

1. Enjoyment and pleasure

4

3

2

1

I can have all of the enjoyment and pleasure that I want

I can have a lot of the enjoyment and pleasure that I want

I can have a little of the enjoyment and pleasure that I want

I cannot have any of the enjoyment and pleasure that I want

1. Independence

4

3

2

1

I am able to be completely independent

I am able to be independent in many things

I am able to be independent in a few things

I am unable to be at all independent

© Joanna Coast & Terry Flynn

**ABOUT YOUR QUALITY OF LIFE IF YOU HAD A DISABILITY**

Now imagine you have a SENSORY OR PHYSICAL DISABILITY. By placing a tick in

ONE box in EACH group below, please indicate which statement would best describe

your quality of life.

1. Love and Friendship

4

3

2

1

I can have all of the love and friendship I want

I can have a lot of the love and friendship I want

I can have a little of the love and friendship I want

I cannot have any of the love and friendship I want

1. Thinking about the future

4

3

2

1

I can think about the future without any concern

I can think about the future with only a little concern

I can only think about the future with some concern

I can only think about the future with a lot of concern

**Tick**

**one**

**box**

**only in**

**each**

**section**

1. Doing things that make you feel valued

4

3

2

1

I am able to do all of the things that make me feel valued

I am able to many of the things that make me feel valued

I am able to do a few of the things that make me feel valued

I am unable to do any of the things that make me feel valued

1. Enjoyment and pleasure

4

3

2

1

I can have all of the enjoyment and pleasure that I want

I can have a lot of the enjoyment and pleasure that I want

I can have a little of the enjoyment and pleasure that I want

I cannot have any of the enjoyment and pleasure that I want

1. Independence

4

3

2

1

I am able to be completely independent

I am able to be independent in many things

I am able to be independent in a few things

I am unable to be at all independent

© Joanna Coast & Terry Flynn

**B Perceptions of Disability**

Lisa is a 50-year-old female and has a physical disability. She was first diagnosed with relapse-remitting multiple sclerosis (MS) 11 years ago. Typical symptoms include fatigue and difficulty with walking.

For the following questions, please circle the number that best corresponds to your views:

**How much do you think this physical disability would affect your life?**

0 1 2 3 4 5 6 7 8 9 10

no affect severely

at all affects my life

**How long do you think this physical disability would continue?**

0 1 2 3 4 5 6 7 8 9 10

a very forever

short time

**How much control would you feel you had over this physical disability?**

0 1 2 3 4 5 6 7 8 9 10

absolutely extreme amount

no control of control

**How much do you think treatment could help this physical disability?**

0 1 2 3 4 5 6 7 8 9 10

not at all extremely

helpful helpful

**How much do you think you would experience associated symptoms from this physical disability?**

0 1 2 3 4 5 6 7 8 9 10

no symptoms many severe

at all symptoms

**How concerned would you be about having this physical disability?**

0 1 2 3 4 5 6 7 8 9 10

not at all extremely

concerned concerned

**How well do you feel you would understand this physical disability?**

0 1 2 3 4 5 6 7 8 9 10

don't understand understand

at all very clearly

**How much would this physical disability affect you emotionally? (e.g. would it make you angry, scared, upset or depressed?**

0 1 2 3 4 5 6 7 8 9 10

not at all extremely

affected affected

emotionally emotionally

**Please list in rank-order the three most important factors that you believe cause physical disability. *The most important causes for me:-***

**1. __________________________________**

**2. __________________________________**

**3. __________________________________**

© All rights reserved. For permission to use the scale please contact: lizbroadbent@clear.net.nz

Hannah is an 86-year-old woman and has a visual impairment. She was first diagnosed with Dry Age-Related Macular Degeneration (AMD) 10 years ago. She has a large blind spot in the centre of her vision and has difficulty recognising faces and reading.

For the following questions, please circle the number that best corresponds to your views:

**How much do you think this sensory disability would affect your life?**

0 1 2 3 4 5 6 7 8 9 10

no affect severely

at all affects my life

**How long do you think this sensory disability would continue?**

0 1 2 3 4 5 6 7 8 9 10

a very forever

short time

**How much control would you feel you have over this sensory disability?**

0 1 2 3 4 5 6 7 8 9 10

absolutely extreme amount

no control of control

**How much do you think treatment could help this sensory disability?**

0 1 2 3 4 5 6 7 8 9 10

not at all extremely

helpful helpful

**How much do you think you would experience associated symptoms from this sensory disability?**

0 1 2 3 4 5 6 7 8 9 10

no symptoms many severe

at all symptoms

**How concerned would you be about having this sensory disability?**

0 1 2 3 4 5 6 7 8 9 10

not at all extremely

concerned concerned

**How well do you feel you would understand this sensory disability?**

0 1 2 3 4 5 6 7 8 9 10

don't understand understand

at all very clearly

**How much would this sensory disability affect you emotionally? (e.g. would it make you angry, scared, upset or depressed?**

0 1 2 3 4 5 6 7 8 9 10

not at all extremely

affected affected

emotionally emotionally

**Please list in rank-order the three most important factors that you believe cause sensory disability. *The most important causes:-***

**1. __________________________________**

**2. __________________________________**

**3. __________________________________**

© All rights reserved. For permission to use the scale please contact: lizbroadbent@clear.net.nz

David is a 12-year-old boy and has a physical disability. He was first diagnosed with Spastic Hemiplegia (a form of Cerebral Palsy) when he was 3 years old. His symptoms include difficulties talking, walking and using his right arm.

For the following questions, please circle the number that best corresponds to your views:

**How much do you think this physical disability would affect your life?**

0 1 2 3 4 5 6 7 8 9 10

no affect severely

at all affects my life

**How long do you do you think this physical disability would continue?**

0 1 2 3 4 5 6 7 8 9 10

a very forever

short time

**How much control do you feel you would have over this physical disability?**

0 1 2 3 4 5 6 7 8 9 10

absolutely extreme

no control amount

of control

**How much do you think treatment could help this physical disability?**

0 1 2 3 4 5 6 7 8 9 10

not at all extremely

helpful helpful

**How much do you think you would experience associated symptoms from this physical disability?**

0 1 2 3 4 5 6 7 8 9 10

no symptoms many

at all severe

symptoms

**How concerned would you be about having this physical disability?**

0 1 2 3 4 5 6 7 8 9 10

not at all extremely

concerned concerned

**How well do you feel you would understand this physical disability?**

0 1 2 3 4 5 6 7 8 9 10

don't understand understand

at all very clearly

**How much would this physical disability affect you emotionally? (e.g. would it make you angry, scared, upset or depressed?**

0 1 2 3 4 5 6 7 8 9 10

not at all extremely

affected affected

emotionally emotionally

**Please list in rank-order the three most important factors that you believe cause learning disability. *The most important causes :-***

**1. __________________________________**

**2. __________________________________**

**3. __________________________________**

© All rights reserved. For permission to use the scale please contact: lizbroadbent@clear.net.nz

**C Perceptions of Disability Thermometer**

Imagine you have the following disability for a prolonged period of your life. Please draw a line from each box to indicate on the thermometer what you think it would be like to have such a disability. Lines can cross. When you have done this please draw a horizontal line across the thermometer to indicate where you think the state of death would be.

**100**

**0**

***Worst imaginable health state***

***Best imaginable***

***health state***

**10**

**60**

**50**

**40**

**30**

**20**

**90**

**80**

**70**

**Child with physical mobility restriction**

you have difficulty walking more than 50 steps unaided

Hearing impairment

you are profoundly deaf, cannot hear voices, TV or radio or traffic

Child with physical mobility restriction

you have severe restrictions on mobility, self-care and daily living

Visual Impairment

you have 30% vision, cannot see faces or read and can move around unfamiliar surroundings with difficulty

**Physical mobility restriction**

you have severe restrictions on mobility, self-care and daily living

**D Perceptions of Disability with adaptations for daily living**

Physical mobility restriction

you have difficulty walking more than 50 steps unaided

Visual Impairment

you can distinguish between light and dark but cannot see faces, read print or move around in unfamiliar surroundings

**Hearing impairment**

you have 30% hearing, you can hear individual voices but cannot distinguish speech from background noise

Imagine you have the following disability with adaptations and/or support to help with daily living. Please draw a line from each box to indicate on the thermometer what you think it would be like to be in such a situation. Lines can cross. Feel free to refer back to your responses to section C. When you have done this please draw a horizontal line across the thermometer to indicate where you think the state of death would be

**100**

**0**

***Worst imaginable health state***

***Best imaginable***

***health state***

**10**

**60**

**50**

**40**

**30**

**20**

**90**

**80**

**70**

**Child with physical mobility restriction**

you can undertake activities of daily living with the aid of a powered wheelchair. You are not mobile without a wheelchair or help from a carer

Hearing impairment

you are profoundly deaf, can lip-read, speak and use sign language. A hearing dog can help with daily life

Visual Impairment

you can distinguish between light and dark, and move around unfamiliar surroundings with a guide dog. You can read via Braille or with an electronic reader

Physical mobility restriction

you can undertake activities of daily living with the aid of a manual wheelchair. You can walk short distances (less than 50 steps) without a wheelchair

Visual Impairment

you have 30% vision and can move around confidently in unfamiliar surroundings with a cane. You can read via large print or with an electronic reader

**Physical mobility restriction**

you can undertake activities of daily living with help from a carer, powered wheelchair or motorised scooter. You are not mobile without a wheelchair or help from a carer

**E The NHS and Disability**

**Hearing impairment**

you have 30% hearing, can hear voices with a hearing aid, amplified telephones, and can lip-read, speak and use sign language

Child with physical mobility restriction

you can undertake activities of daily living with the aid of a manual wheelchair. You can walk short distances (less than 50 steps) without a wheelchair

The NHS is tax funded, has limited resources and must try to meet the needs of patients. Please indicate whether you agree or disagree with the following statements:

1. Older people should take priority **strongly agree don’t disagree strongly**

over younger people in gaining NHS **agree know disagree**

treatment. ❑ ❑ ❑ ❑ ❑

2. Children should take priority **strongly agree don’t disagree strongly**

over older people in gaining NHS **agree know disagree**

treatment. ❑ ❑ ❑ ❑ ❑

3. Disabled people should take priority **strongly agree don’t disagree strongly**

over people without disability in gaining **agree know disagree**

NHS treatment. ❑ ❑ ❑ ❑ ❑

4. Disabled children should take priority **strongly agree don’t disagree strongly**

over older disabled people and people **agree know disagree**

without disability in gaining NHS treatment. ❑ ❑ ❑ ❑ ❑

5. In general, disabled people, even if they  **strongly agree don’t disagree strongly**

are not ill, have a poorer health related **agree know disagree**

quality of life than people without ❑ ❑ ❑ ❑ ❑

disability.

6. In general, with financial, practical and  **strongly agree don’t disagree strongly**

environmental support and adaptation, **agree know disagree**

people with disabilities can have as good ❑ ❑ ❑ ❑ ❑

a health related quality of life as people

without disability.

**F General Questions**

*Because all replies are anonymous, it will help to understand your answers better if we have a little background data from everyone, as covered in the following questions:*

1. What is your age in years?

2. Are you: … Male  or Female  *PLEASE TICK*

*APPROPRIATE BOX*

3. Are you:

a current smoker 

an ex-smoker  *PLEASE TICK*

a never smoker  *APPROPRIATE BOX*

4. Have you ever experienced serious illness …

in you yourself? Yes  No  *PLEASE TICK*

in your family? Yes  No  *APPROPRIATE*

in caring for others? Yes  No  *BOXES*

5. Do you consider yourself to have a disability? Yes  No 

If yes, please briefly describe your disability _____________________________________

6. Do you have a family member or close Yes  No 

friend who has a disability?

If yes, please briefly describe their disability ____________________________________

**7. If yes to 4 OR 5, please tick if any assistive technology is used to support disability**

|  | **Yourself** | **Family /**  **Close friend** |
| --- | --- | --- |
| **Mobility aids** *(e.g. wheelchairs, canes, walkers etc.)* |  |  |
| **Communication aids** *(e.g. voice amplifier, communication boards etc.)* |  |  |
| **Environmental** *(e.g. home modifications, computer access aids, environmental controls)* |  |  |
| **Vehicle aids** *(e.g. modified control system, transfer adaptations etc.)* |  |  |
| **Medical aids** *(e.g. continence aids, functional electrical stimulation etc.)* |  |  |
| **Memory aids** *(e.g. daily planner, electronic reminders etc.)* |  |  |
| **Kitchen aids** *(e.g. specialised cooking aids, specialised eating aids etc.)* |  |  |
| **Bathroom aids** *(e.g. raised toilet/seat, grab bars, commode etc.)* |  |  |
| **Telecare** *(e.g. fall detectors, alarm systems, medication devices etc.)* |  |  |
| **Other** *(please state)* |  |  |
