# Supplementary material for: Perceptions of the impact of disability and impairment on health, quality of life and capability
Source: BMC Res Notes. 2019 May 24;12:287. doi: 10.1186/s13104-019-4324-y (PMC6534923; doi:10.1186/s13104-019-4324-y)
Supplement: Supplementary file 2 — Additional file 2: Table S1. Perception of EQ-VAS health status in states of visual, hearing and mobility impairment, and the effect of adaptation. This table shows the effect of adaptation on the perceived health status (measured using EQ-VAS) of different states of visual, hearing and mobility impairment. [file 13104_2019_4324_MOESM2_ESM.docx]

*Table S1: Perception of EQ-VAS health status in states of visual, hearing and mobility impairment, and the effect of adaptation*

|  |  | **Mean*** | **SD** | **Range** | **Median** |
| --- | --- | --- | --- | --- | --- |
| **Visual Impairment** | Severe | 37.40 | 20.74 | 0 to 90 | 40 |
|  | Severe adapted | 51.76 | 21.31 | 0 to 100 | 50 |
|  | Moderate | 38.77 | 20.13 | 0 to 90 | 40 |
|  | Moderate adapted | 56.19 | 21.77 | 1 to 100 | 60 |
| **Hearing impairment** | Severe | 38.12 | 21.51 | 0 to 90 | 40 |
|  | Severe adapted | 55.65 | 20.24 | 10 to 100 | 60 |
|  | Moderate | 53.65 | 18.65 | 10 to 100 | 55 |
|  | Moderate adapted | 60.51 | 19.50 | 10 to 100 | 60 |
| **Mobility impairment** | Severe | 25.08 | 19.22 | 0 to 85 | 20 |
|  | Severe adapted | 46.68 | 18.94 | 0 to 100 | 50 |
|  | Moderate | 39.13 | 19.78 | 0 to 82 | 40 |
|  | Moderate adapted | 54.24 | 20.93 | 10 to 100 | 55 |

**EQ-VAS scale: 0=worst imaginable health state / 100=best imaginable health state*
