# Supplementary material for: Perceptions of the impact of disability and impairment on health, quality of life and capability
Source: BMC Res Notes. 2019 May 24;12:287. doi: 10.1186/s13104-019-4324-y (PMC6534923; doi:10.1186/s13104-019-4324-y)
Supplement: Supplementary file 3 — Additional file 3: Table S2. Individual item score proportions (%) on the ICECAP-O (personal and perceived results). This table shows the difference between perceived (i.e. a hypothetical state of generic impairment) and personal item scores on the ICECAP-O. [file 13104_2019_4324_MOESM3_ESM.docx]

*Table S2: Individual item score proportions (%) on the ICECAP-O (personal and perceived results)*

|  |  | **Attachment** | **Security** | **Role** | **Enjoyment** | **Control** |
| --- | --- | --- | --- | --- | --- | --- |
| **Personal*** | **1** | 0.66 | 7.28 | 2.65 | 0.00 | 0.00 |
|  | **2** | 12.58 | 27.81 | 18.54 | 17.22 | 3.97 |
|  | **3** | 58.94 | 48.34 | 49.01 | 49.67 | 43.71 |
|  | **4** | 27.81 | 16.56 | 29.80 | 33.11 | 52.32 |
|  | **Mean** | 3.14 | 2.74 | 3.06 | 3.16 | 3.48 |
|  | **SD** | 0.64 | 0.82 | 0.77 | 0.69 | 0.58 |
| **Perceived*** | **1** | 1.33 | 26.67 | 8.67 | 5.33 | 8.67 |
|  | **2** | 26.67 | 44.00 | 45.33 | 44.00 | 63.33 |
|  | **3** | 50.67 | 24.00 | 32.67 | 43.33 | 20.67 |
|  | **4** | 21.33 | 5.33 | 13.33 | 7.33 | 7.33 |
|  | **Mean** | 2.92 | 2.08 | 2.51 | 2.53 | 2.27 |
|  | **SD** | 0.73 | 0.85 | 0.83 | 0.71 | 0.72 |

**ICECAP-O item scale: 1=lowest wellbeing item score / 4=highest wellbeing item score*
