# Supplementary material for: Perceptions of the impact of disability and impairment on health, quality of life and capability
Source: BMC Res Notes. 2019 May 24;12:287. doi: 10.1186/s13104-019-4324-y (PMC6534923; doi:10.1186/s13104-019-4324-y)
Supplement: Supplementary file 4 — Additional file 4: Table S3. Individual item score proportions (%) on the B-IPQ (mobility impairment scenario). This table shows perceived individual item scores on the B-IPQ for a hypothetical state of mobility impairment. [file 13104_2019_4324_MOESM4_ESM.docx]

*Table S3: Individual item score proportions (%) on the B-IPQ (mobility impairment scenario)*

| **Item scores*** | **Consequences** | **Timeline** | **Personal Control** | **Treatment Control** | **Identity** | **Concern** | **Coherence** | **Emotional Representation** |
| --- | --- | --- | --- | --- | --- | --- | --- | --- |
| 0 | 0.00 | 0.00 | 2.00 | 6.00 | 0.00 | 0.00 | 5.33 | 0.67 |
| 1 | 0.67 | 0.00 | 0.00 | 0.67 | 0.00 | 1.33 | 6.00 | 0.00 |
| 2 | 1.33 | 0.00 | 4.67 | 13.33 | 0.67 | 0.67 | 13.33 | 2.00 |
| 3 | 2.67 | 0.00 | 4.00 | 17.33 | 4.00 | 1.33 | 12.67 | 2.00 |
| 4 | 2.00 | 0.67 | 3.33 | 19.33 | 3.33 | 2.00 | 14.67 | 2.00 |
| 5 | 2.67 | 6.00 | 14.00 | 20.67 | 7.33 | 7.33 | 12.67 | 4.67 |
| 6 | 16.00 | 4.67 | 12.67 | 11.33 | 26.00 | 10.67 | 18.00 | 17.33 |
| 7 | 28.00 | 10.67 | 17.33 | 8.00 | 33.33 | 19.33 | 8.67 | 22.00 |
| 8 | 26.67 | 16.00 | 26.00 | 2.00 | 17.33 | 17.33 | 5.33 | 17.33 |
| 9 | 11.33 | 18.67 | 8.00 | 1.33 | 6.00 | 15.33 | 2.67 | 16.67 |
| 10 | 8.67 | 43.33 | 8.00 | 0.00 | 2.00 | 24.67 | 0.67 | 15.33 |
| **MEAN** | **7.27** | **8.65** | **6.62** | **4.12** | **6.65** | **7.74** | **4.35** | **7.41** |
| **SD** | 1.70 | 1.57 | 2.23 | 1.94 | 1.47 | 1.98 | 2.31 | 1.94 |

**1=least threatening illness perception / 10=most threatening illness perception*
