# Supplementary material for: Perceptions of the impact of disability and impairment on health, quality of life and capability
Source: BMC Res Notes. 2019 May 24;12:287. doi: 10.1186/s13104-019-4324-y (PMC6534923; doi:10.1186/s13104-019-4324-y)
Supplement: Supplementary file 5 — Additional file 5: Table S4. Individual item score proportions (%) on the B-IPQ (visual impairment scenario). This table shows perceived individual item scores on the B-IPQ for a hypothetical state of visual impairment. [file 13104_2019_4324_MOESM5_ESM.docx]

*Table S4: Individual item score proportions (%) on the B-IPQ (visual impairment scenario)*

| **Item scores*** | **Consequences** | **Timeline** | **Personal Control** | **Treatment Control** | **Identity** | **Concern** | **Coherence** | **Emotional Representation** |
| --- | --- | --- | --- | --- | --- | --- | --- | --- |
| 0 | 0.00 | 0.00 | 1.32 | 2.65 | 0.66 | 0.00 | 2.65 | 0.66 |
| 1 | 0.66 | 0.00 | 1.99 | 1.32 | 0.66 | 1.32 | 10.60 | 0.00 |
| 2 | 0.00 | 0.00 | 4.64 | 7.28 | 4.64 | 0.00 | 12.58 | 0.66 |
| 3 | 1.32 | 0.00 | 3.97 | 12.58 | 8.61 | 1.99 | 13.91 | 1.99 |
| 4 | 2.65 | 0.00 | 4.64 | 17.88 | 7.28 | 1.99 | 16.56 | 3.97 |
| 5 | 1.32 | 3.31 | 3.31 | 15.23 | 16.56 | 5.96 | 11.92 | 7.28 |
| 6 | 9.27 | 3.31 | 6.62 | 9.27 | 11.92 | 8.61 | 10.60 | 11.26 |
| 7 | 19.87 | 5.30 | 15.23 | 13.91 | 20.53 | 12.58 | 9.93 | 13.91 |
| 8 | 23.84 | 12.58 | 21.85 | 10.60 | 16.56 | 14.57 | 7.95 | 20.53 |
| 9 | 18.54 | 21.19 | 16.56 | 5.96 | 3.97 | 19.87 | 2.65 | 19.21 |
| 10 | 22.52 | 54.30 | 19.87 | 3.31 | 8.61 | 33.11 | 0.66 | 20.53 |
| **MEAN** | **7.99** | **9.08** | **7.27** | **5.26** | **6.17** | **8.11** | **4.30** | **7.67** |
| **SD** | 1.70 | 1.31 | 2.53 | 2.36 | 2.23 | 2.00 | 2.36 | 1.99 |

**1=least threatening illness perception / 10=most threatening illness perception*
